# 5-(5′-Fluoro-2′-meth­oxy­biphenyl-3-yl)-1,3,4-oxa­diazol-2-amine

**DOI:** 10.1107/S1600536813031206

**Published:** 2013-11-20

**Authors:** M. K. Usha, G. C. Ramaprasad, Balakrishna Kalluraya, Rajni Kant, Vivek K. Gupta, D. Revannasiddaiah

**Affiliations:** a Department of Studies in Physics, University of Mysore, Manasagangotri, Mysore 570 006, India; b Department of Studies in Chemistry, Mangalore University, Mangalagangotri, Mangalore 574 199, India; c Post-Graduate Department of Physics & Electronics, University of Jammu, Jammu Tawi 180 006, India

## Abstract

In the title compound, C_15_H_12_FN_3_O_2_, the dihedral angles between the central benzene ring and the pendant benzene and oxa­diazole rings are 45.05 (13) and 15.60 (14)°, respectively. The C atom of the meth­oxy group is roughly coplanar with its attached ring [displacement = 0.178 (4) Å]. In the crystal, N—H⋯N hydrogen bonds link the mol­ecules into [010] chains. Weak C—H⋯π inter­actions are also observed.

## Related literature

For background to the title compound, see: Ainsworth (1965 ▶); Paik *et al.* (2002 ▶); Kulkarni *et al.* (2004 ▶). For a related structure, see: Zheng *et al.* (2012 ▶).
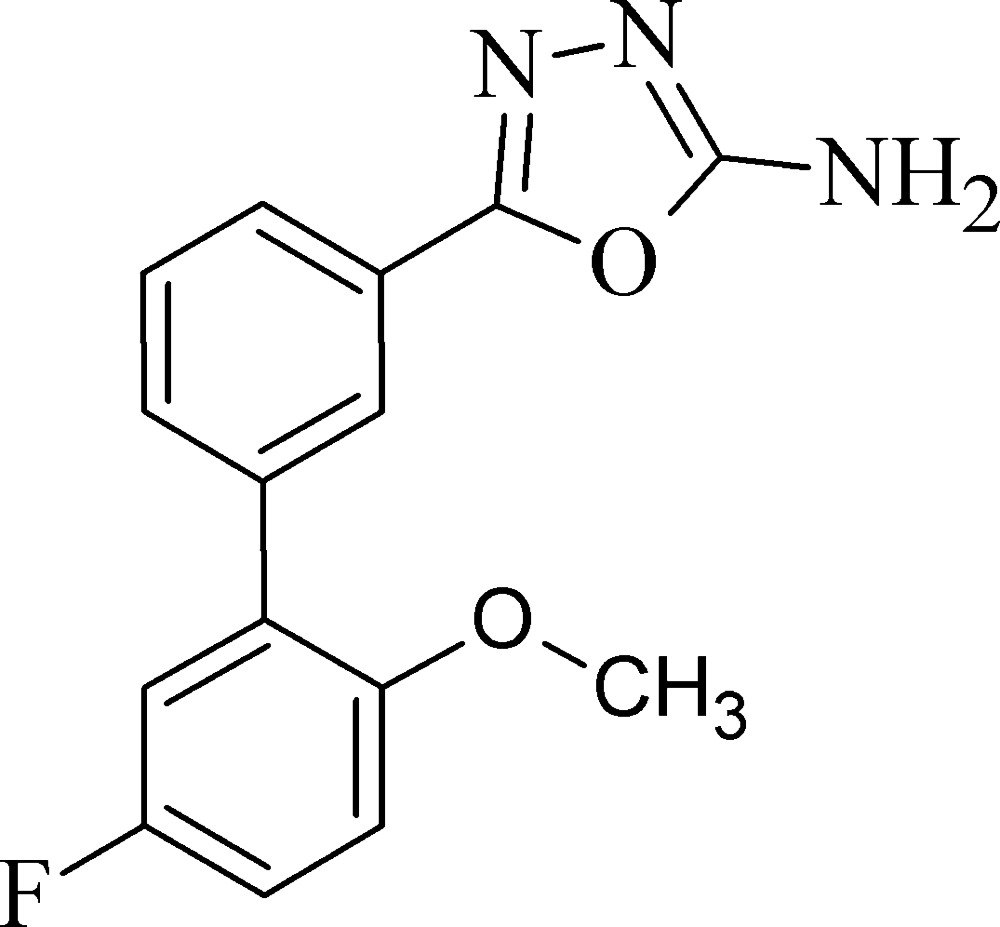


## Experimental

### 

#### Crystal data

C_15_H_12_FN_3_O_2_
*M* *_r_* = 285.28
Monoclinic, 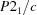

*a* = 12.9105 (9) Å
*b* = 6.1738 (4) Å
*c* = 16.9255 (11) Å
β = 90.341 (7)°
*V* = 1349.05 (16) Å^3^
*Z* = 4
Mo *K*α radiation
μ = 0.11 mm^−1^
*T* = 293 K
0.30 × 0.20 × 0.20 mm

#### Data collection

Oxford Diffraction Xcalibur Sapphire3 CCD diffractometer
Absorption correction: multi-scan (*CrysAlis RED*; Oxford Diffraction, 2010 ▶) *T*_min_ = 0.806, *T*_max_ = 1.000
5027 measured reflections
2650 independent reflections
1382 reflections with *I* > 2σ(*I*)
*R*_int_ = 0.048

#### Refinement

*R*[*F* ^2^ > 2σ(*F* ^2^)] = 0.054
*wR*(*F* ^2^) = 0.135
*S* = 1.01
2650 reflections
191 parameters
H-atom parameters constrained
Δρ_max_ = 0.18 e Å^−3^
Δρ_min_ = −0.18 e Å^−3^

### 

Data collection: *CrysAlis PRO* (Oxford Diffraction, 2010 ▶); cell refinement: *CrysAlis PRO*; data reduction: *CrysAlis PRO*; program(s) used to solve structure: *SHELXS97* (Sheldrick, 2008 ▶); program(s) used to refine structure: *SHELXL97* (Sheldrick, 2008 ▶); molecular graphics: *ORTEP-3 for Windows* (Farrugia, 2012 ▶) and *Mercury* (Macrae *et al.*, 2008 ▶); software used to prepare material for publication: *PLATON* (Spek, 2009 ▶).

## Supplementary Material

Crystal structure: contains datablock(s) I, New_Global_Publ_Block. DOI: 10.1107/S1600536813031206/hb7155sup1.cif

Structure factors: contains datablock(s) I. DOI: 10.1107/S1600536813031206/hb7155Isup2.hkl

Supplementary material file. DOI: 10.1107/S1600536813031206/hb7155Isup3.cml

Additional supplementary materials:  crystallographic information; 3D view; checkCIF report

## Figures and Tables

**Table 1:** Hydrogen-bond geometry (Å, °) *Cg*3 is the centroid of the C13–C18 ring.

*D*—H⋯*A*	*D*—H	H⋯*A*	*D*⋯*A*	*D*—H⋯*A*
N6—H6*A*⋯N3^i^	0.86	2.13	2.972 (3)	166
N6—H6*B*⋯N4^ii^	0.86	2.29	3.118 (3)	161
C17—H17⋯*Cg*3^iii^	0.93	2.72	3.53	146

Symmetry codes: (i) 
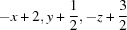
; (ii) 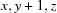; (iii) 
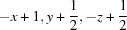
.

**Fig. 1.**
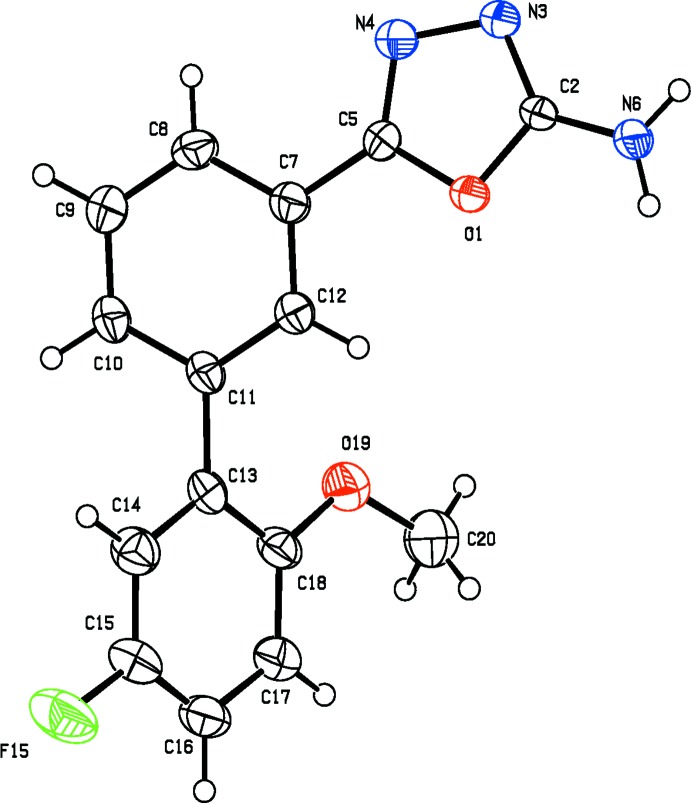
ORTEP view of the molecule with displacement ellipsoids drawn at the 40% probability level.

**Fig. 2.**
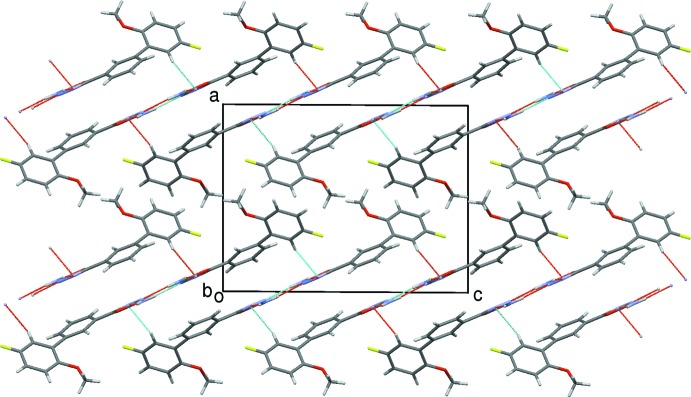
The packing arrangement of molecules viewed along the a axis.

## supplementary crystallographic information

### 1. Comment

The title compound, C_15_H_12_FN_3_O_2_, a derivative of 1,3,4 oxadiazole (Ainsworth, 1965), has a wide variety of uses, particularly as a bioactive compound in medicine and agriculture, as a dye stuff, and used in UV absorbing and fluorescent materials, heat resistant polymers and scintillators (Paik *et al.*, 2002; Kulkarni *et al.*, 2004). As part of our studies in this area, we now report the structure of the title compound.

The bond distances in the title compound are comparable to the closely related structure 5-(4-Methylphenyl)-1,3,4-oxadiazol-2-amine (Zheng *et al.*, 2012). The oxadiazole ring A makes dihedral angles of 15.64 (9)° and 55.84 (1)° respectively, with the phenyl rings B and C. The dihedral angle between the phenyl ring B and ring C is 45.19 (1)°. Classical N6—H6A···N3 and N6—H6B···N4 hydrogen bonds link the adjacent molecules into [010] chains. Weak C—H···π interactions are also observed.

### 2. Experimental

To a solution of 5'-fluoro-2'-methoxybiphenyl-3-carbohydrazide (3.84 mmol)in 1,4-dioxane (10 ml) cyanogen bromide (3.84 mmol) was added, followed by a solution of sodium bicarbonate (3.84 mmol) in water (10 ml). The resulting mixture was stirred at room temperature for 2 h. The reaction mixture was taken in ethyl acetate (100 ml), washed with water (20 ml) followed by saturated sodium chloride solution (20 ml) and dried over sodium sulfate. The resulting solution was concentrated and purified by column chromatography [30–40% ethyl acetate in petroleum ether] to afford the title compound (M.P. 223–226°C).

### 3. Refinement

The H atoms were positioned geometrically and were refined as riding on their parent C and N atoms, with C—H distances of 0.88–0.95 Å, N—H distance of 0.86 Å and with *U*_iso_(H) = 1.2*U*_eq_(C).

### Crystal data

**Table:**

C_15_H_12_FN_3_O_2_	*F*(000) = 592
*M**_r_* = 285.28	*D*_x_ = 1.405 Mg m^−^^3^
Monoclinic, *P*2_1_/*c*	Mo *K*α radiation, λ = 0.71073 Å
Hall symbol: -P 2ybc	Cell parameters from 1316 reflections
*a* = 12.9105 (9) Å	θ = 4.1–28.0°
*b* = 6.1738 (4) Å	µ = 0.11 mm^−^^1^
*c* = 16.9255 (11) Å	*T* = 293 K
β = 90.341 (7)°	Block, colourless
*V* = 1349.05 (16) Å^3^	0.30 × 0.20 × 0.20 mm
*Z* = 4	

### Data collection

**Table:**

Oxford Diffraction Xcalibur Sapphire3 CCD diffractometer	2650 independent reflections
Radiation source: fine-focus sealed tube	1382 reflections with *I* > 2σ(*I*)
Graphite monochromator	*R*_int_ = 0.048
Detector resolution: 16.1049 pixels mm^-1^	θ_max_ = 26.0°, θ_min_ = 3.5°
ω scans	*h* = −8→15
Absorption correction: multi-scan (*CrysAlis RED*; Oxford Diffraction, 2010)	*k* = −7→4
*T*_min_ = 0.806, *T*_max_ = 1.000	*l* = −17→20
5027 measured reflections	

### Refinement

**Table:**

Refinement on *F*^2^	Primary atom site location: structure-invariant direct methods
Least-squares matrix: full	Secondary atom site location: difference Fourier map
*R*[*F*^2^ > 2σ(*F*^2^)] = 0.054	Hydrogen site location: inferred from neighbouring sites
*wR*(*F*^2^) = 0.135	H-atom parameters constrained
*S* = 1.01	*w* = 1/[σ^2^(*F*_o_^2^) + (0.0316*P*)^2^] where *P* = (*F*_o_^2^ + 2*F*_c_^2^)/3
2650 reflections	(Δ/σ)_max_ = 0.001
191 parameters	Δρ_max_ = 0.18 e Å^−^^3^
0 restraints	Δρ_min_ = −0.18 e Å^−^^3^

### Special details

**Table:**

Experimental. *CrysAlis PRO*, Agilent Technologies, Version 1.171.36.28 (release 01–02-2013 CrysAlis171. NET) (compiled Feb 1 2013,16:14:44) Empirical absorption correction using spherical harmonics, implemented in SCALE3 ABSPACK scaling algorithm.
Geometry. All e.s.d.'s (except the e.s.d. in the dihedral angle between two l.s. planes) are estimated using the full covariance matrix. The cell e.s.d.'s are taken into account individually in the estimation of e.s.d.'s in distances, angles and torsion angles; correlations between e.s.d.'s in cell parameters are only used when they are defined by crystal symmetry. An approximate (isotropic) treatment of cell e.s.d.'s is used for estimating e.s.d.'s involving l.s. planes.
Refinement. Refinement of *F*^2^ against ALL reflections. The weighted *R*-factor *wR* and goodness of fit *S* are based on *F*^2^, conventional *R*-factors *R* are based on *F*, with *F* set to zero for negative *F*^2^. The threshold expression of *F*^2^ > σ(*F*^2^) is used only for calculating *R*-factors(gt) *etc*. and is not relevant to the choice of reflections for refinement. *R*-factors based on *F*^2^ are statistically about twice as large as those based on *F*, and *R*- factors based on ALL data will be even larger.

### Fractional atomic coordinates and isotropic or equivalent isotropic displacement parameters (Å^2^)

**Table:**

	*x*	*y*	*z*	*U*_iso_*/*U*_eq_	
O1	0.89022 (14)	0.0443 (3)	0.56943 (11)	0.0411 (5)	
C2	0.9336 (2)	0.0508 (4)	0.64301 (15)	0.0357 (7)	
N3	0.95451 (18)	−0.1405 (3)	0.67077 (13)	0.0435 (6)	
N4	0.92173 (18)	−0.2865 (3)	0.61121 (14)	0.0440 (6)	
C5	0.8848 (2)	−0.1745 (4)	0.55387 (16)	0.0361 (7)	
N6	0.94739 (18)	0.2434 (3)	0.67575 (14)	0.0522 (7)	
H6A	0.9738	0.2534	0.7224	0.063*	
H6B	0.9299	0.3586	0.6504	0.063*	
C7	0.8429 (2)	−0.2443 (4)	0.47839 (16)	0.0394 (7)	
C8	0.8602 (2)	−0.4578 (4)	0.45329 (17)	0.0454 (8)	
H8	0.8959	−0.5549	0.4854	0.054*	
C9	0.8235 (2)	−0.5213 (4)	0.38043 (18)	0.0480 (8)	
H9	0.8363	−0.6615	0.3628	0.058*	
C10	0.7682 (2)	−0.3804 (4)	0.33300 (17)	0.0466 (8)	
H10	0.7439	−0.4268	0.2840	0.056*	
C11	0.7485 (2)	−0.1694 (4)	0.35787 (16)	0.0388 (7)	
C12	0.7856 (2)	−0.1043 (4)	0.43139 (16)	0.0405 (7)	
H12	0.7718	0.0352	0.4493	0.049*	
C13	0.6929 (2)	−0.0182 (4)	0.30377 (17)	0.0411 (7)	
C14	0.7207 (2)	−0.0126 (5)	0.22425 (18)	0.0506 (8)	
H14	0.7731	−0.1027	0.2059	0.061*	
C15	0.6709 (2)	0.1257 (5)	0.17307 (18)	0.0524 (9)	
F15	0.70017 (16)	0.1262 (3)	0.09598 (11)	0.0846 (7)	
C16	0.5951 (3)	0.2629 (5)	0.19672 (19)	0.0531 (9)	
H16	0.5637	0.3574	0.1611	0.064*	
C17	0.5654 (2)	0.2585 (5)	0.27592 (17)	0.0517 (9)	
H17	0.5122	0.3480	0.2932	0.062*	
C18	0.6147 (2)	0.1221 (4)	0.32879 (17)	0.0441 (8)	
O19	0.58667 (17)	0.1049 (3)	0.40644 (12)	0.0614 (7)	
C20	0.5209 (3)	0.2668 (6)	0.4382 (2)	0.0936 (14)	
H20A	0.5506	0.4071	0.4290	0.140*	
H20B	0.5134	0.2441	0.4940	0.140*	
H20C	0.4541	0.2588	0.4130	0.140*	

### Atomic displacement parameters (Å^2^)

**Table:**

	*U*^11^	*U*^22^	*U*^33^	*U*^12^	*U*^13^	*U*^23^
O1	0.0593 (13)	0.0289 (9)	0.0351 (11)	0.0003 (9)	−0.0166 (10)	0.0016 (9)
C2	0.0461 (17)	0.0308 (14)	0.0301 (16)	0.0027 (12)	−0.0122 (14)	0.0017 (13)
N3	0.0615 (17)	0.0327 (12)	0.0360 (15)	0.0038 (11)	−0.0156 (13)	0.0011 (12)
N4	0.0553 (16)	0.0352 (13)	0.0412 (15)	0.0026 (11)	−0.0122 (14)	0.0020 (11)
C5	0.0457 (17)	0.0279 (14)	0.0345 (17)	−0.0006 (12)	−0.0035 (14)	−0.0008 (13)
N6	0.0814 (19)	0.0304 (12)	0.0444 (16)	0.0014 (12)	−0.0292 (15)	−0.0041 (12)
C7	0.0481 (17)	0.0339 (15)	0.0362 (18)	−0.0033 (13)	−0.0061 (15)	0.0014 (14)
C8	0.0564 (19)	0.0326 (14)	0.0471 (19)	−0.0002 (14)	−0.0083 (16)	0.0077 (14)
C9	0.061 (2)	0.0363 (15)	0.047 (2)	−0.0004 (14)	−0.0050 (17)	−0.0040 (15)
C10	0.060 (2)	0.0403 (17)	0.0391 (18)	−0.0048 (14)	−0.0127 (16)	−0.0072 (14)
C11	0.0448 (18)	0.0384 (15)	0.0331 (17)	−0.0026 (13)	−0.0104 (15)	−0.0035 (13)
C12	0.0461 (17)	0.0359 (15)	0.0393 (18)	−0.0002 (13)	−0.0103 (15)	−0.0027 (14)
C13	0.0440 (18)	0.0432 (17)	0.0359 (18)	−0.0017 (13)	−0.0122 (15)	−0.0060 (14)
C14	0.0465 (19)	0.058 (2)	0.047 (2)	0.0030 (15)	−0.0033 (16)	0.0008 (17)
C15	0.053 (2)	0.066 (2)	0.0376 (19)	−0.0013 (17)	−0.0043 (17)	0.0113 (17)
F15	0.0898 (16)	0.1211 (17)	0.0430 (12)	0.0135 (13)	0.0050 (12)	0.0220 (12)
C16	0.063 (2)	0.052 (2)	0.044 (2)	−0.0017 (17)	−0.0132 (18)	0.0107 (17)
C17	0.056 (2)	0.0550 (19)	0.044 (2)	0.0098 (16)	−0.0097 (17)	0.0001 (17)
C18	0.0494 (19)	0.0460 (17)	0.0367 (18)	−0.0024 (14)	−0.0126 (16)	0.0019 (15)
O19	0.0755 (17)	0.0654 (15)	0.0433 (14)	0.0219 (12)	−0.0017 (13)	−0.0034 (12)
C20	0.131 (4)	0.092 (3)	0.059 (3)	0.044 (3)	0.005 (3)	−0.012 (2)

### Geometric parameters (Å, º)

**Table:**

O1—C2	1.363 (3)	C11—C13	1.490 (4)
O1—C5	1.378 (3)	C12—H12	0.9300
C2—N3	1.299 (3)	C13—C14	1.396 (4)
C2—N6	1.323 (3)	C13—C18	1.398 (4)
N3—N4	1.415 (3)	C14—C15	1.374 (4)
N4—C5	1.281 (3)	C14—H14	0.9300
C5—C7	1.450 (3)	C15—C16	1.356 (4)
N6—H6A	0.8600	C15—F15	1.361 (3)
N6—H6B	0.8600	C16—C17	1.397 (4)
C7—C12	1.386 (3)	C16—H16	0.9300
C7—C8	1.403 (3)	C17—C18	1.381 (4)
C8—C9	1.375 (4)	C17—H17	0.9300
C8—H8	0.9300	C18—O19	1.369 (3)
C9—C10	1.380 (4)	O19—C20	1.420 (3)
C9—H9	0.9300	C20—H20A	0.9600
C10—C11	1.393 (3)	C20—H20B	0.9600
C10—H10	0.9300	C20—H20C	0.9600
C11—C12	1.390 (3)		
			
C2—O1—C5	102.9 (2)	C7—C12—H12	119.6
N3—C2—N6	129.7 (3)	C11—C12—H12	119.6
N3—C2—O1	112.8 (2)	C14—C13—C18	117.9 (3)
N6—C2—O1	117.5 (2)	C14—C13—C11	118.8 (3)
C2—N3—N4	105.1 (2)	C18—C13—C11	123.3 (3)
C5—N4—N3	107.7 (2)	C15—C14—C13	120.1 (3)
N4—C5—O1	111.5 (2)	C15—C14—H14	120.0
N4—C5—C7	130.0 (2)	C13—C14—H14	120.0
O1—C5—C7	118.6 (2)	C16—C15—F15	119.1 (3)
C2—N6—H6A	120.0	C16—C15—C14	122.6 (3)
C2—N6—H6B	120.0	F15—C15—C14	118.3 (3)
H6A—N6—H6B	120.0	C15—C16—C17	118.3 (3)
C12—C7—C8	119.9 (3)	C15—C16—H16	120.9
C12—C7—C5	121.0 (2)	C17—C16—H16	120.9
C8—C7—C5	119.1 (2)	C18—C17—C16	120.4 (3)
C9—C8—C7	119.0 (3)	C18—C17—H17	119.8
C9—C8—H8	120.5	C16—C17—H17	119.8
C7—C8—H8	120.5	O19—C18—C17	123.1 (3)
C8—C9—C10	121.1 (3)	O19—C18—C13	116.0 (3)
C8—C9—H9	119.5	C17—C18—C13	120.8 (3)
C10—C9—H9	119.5	C18—O19—C20	118.1 (3)
C9—C10—C11	120.6 (3)	O19—C20—H20A	109.5
C9—C10—H10	119.7	O19—C20—H20B	109.5
C11—C10—H10	119.7	H20A—C20—H20B	109.5
C12—C11—C10	118.6 (3)	O19—C20—H20C	109.5
C12—C11—C13	122.1 (2)	H20A—C20—H20C	109.5
C10—C11—C13	119.3 (2)	H20B—C20—H20C	109.5
C7—C12—C11	120.9 (2)		
			
C5—O1—C2—N3	0.6 (3)	C10—C11—C12—C7	1.3 (4)
C5—O1—C2—N6	−178.7 (2)	C13—C11—C12—C7	−175.8 (3)
N6—C2—N3—N4	178.8 (3)	C12—C11—C13—C14	133.5 (3)
O1—C2—N3—N4	−0.4 (3)	C10—C11—C13—C14	−43.7 (4)
C2—N3—N4—C5	0.0 (3)	C12—C11—C13—C18	−45.5 (4)
N3—N4—C5—O1	0.4 (3)	C10—C11—C13—C18	137.3 (3)
N3—N4—C5—C7	178.7 (3)	C18—C13—C14—C15	−0.8 (4)
C2—O1—C5—N4	−0.6 (3)	C11—C13—C14—C15	−179.8 (2)
C2—O1—C5—C7	−179.2 (2)	C13—C14—C15—C16	1.0 (5)
N4—C5—C7—C12	165.3 (3)	C13—C14—C15—F15	−179.9 (3)
O1—C5—C7—C12	−16.4 (4)	F15—C15—C16—C17	179.4 (3)
N4—C5—C7—C8	−14.2 (5)	C14—C15—C16—C17	−1.6 (5)
O1—C5—C7—C8	164.1 (2)	C15—C16—C17—C18	1.9 (5)
C12—C7—C8—C9	2.8 (4)	C16—C17—C18—O19	−177.6 (3)
C5—C7—C8—C9	−177.7 (3)	C16—C17—C18—C13	−1.8 (5)
C7—C8—C9—C10	−1.6 (4)	C14—C13—C18—O19	177.3 (3)
C8—C9—C10—C11	0.3 (4)	C11—C13—C18—O19	−3.7 (4)
C9—C10—C11—C12	−0.1 (4)	C14—C13—C18—C17	1.2 (4)
C9—C10—C11—C13	177.2 (3)	C11—C13—C18—C17	−179.8 (3)
C8—C7—C12—C11	−2.7 (4)	C17—C18—O19—C20	−14.6 (4)
C5—C7—C12—C11	177.8 (3)	C13—C18—O19—C20	169.5 (3)

### Hydrogen-bond geometry (Å, º)

Cg3 is the centroid of the C13–C18 ring.

**Table:**

*D*—H···*A*	*D*—H	H···*A*	*D*···*A*	*D*—H···*A*
N6—H6*A*···N3^i^	0.86	2.13	2.972 (3)	166
N6—H6*B*···N4^ii^	0.86	2.29	3.118 (3)	161
C17—H17···*Cg*3^iii^	0.93	2.72	3.53	146

Symmetry codes: (i) −*x*+2, *y*+1/2, −*z*+3/2; (ii) *x*, *y*+1, *z*; (iii) −*x*+1, *y*+1/2, −*z*+1/2.
